# First Report and Complete Genome Sequence of Alfalfa Enamovirus from Sudan

**DOI:** 10.1128/genomeA.00531-17

**Published:** 2017-07-06

**Authors:** Lev G. Nemchinov, Samuel C. Grinstead, Dimitre S. Mollov

**Affiliations:** a USDA-ARS, Molecular Plant Pathology Laboratory, Beltsville, Maryland, USA; b USDA-ARS, National Germplasm Recourses Laboratory, Beltsville, Maryland, USA

## Abstract

We report the complete genome sequence of a viral pathogen detected in alfalfa samples from Sudan and provisionally named alfalfa enamovirus 2 (AEV-2). Based on high nucleotide and amino acid identities, AEV-2 represents a strain of a newly discovered alfalfa enamovirus 1 that has only been described in Argentina.

## GENOME ANNOUNCEMENT

The full-genome sequence of a viral pathogen, provisionally named alfalfa enamovirus 2 (AEV-2), was determined by Illumina RNA sequencing of a total RNA extract from an alfalfa sample originating from Sudan. Ambiguous nucleotides in the consensus assembly and identity of the predicted virus were clarified and confirmed by reverse transcription-PCR (RT-PCR) using primers derived from the Illumina-generated sequence, cloning, and sequencing.

The AEV-2 genome consists of 5,729 nucleotides and contains five open reading frames (ORFs): ORF0 (nucleotides [nt] 181 to 1092), encoding a putative RNA-silencing suppressor; ORF1 (nt 263 to 2557), expressed by a ribosomal leaky scanning mechanism and containing a serine-like protease domain; ORF2 (nt 2017 to 3819), translated by a -1 ribosomal frameshift (nt 2018→2017) from ORF1 and incorporating a core domain of an RNA-directed RNA polymerase (RdRp); ORF3 (nt 4034 to 4603), encoding a putative coat protein (CP); and ORF5 (nt 4034 to 5530), encoding a projected aphid transmission subunit (ATS) translated as an in-frame readthrough extension of ORF3. After translation, ORF1 and ORF2 are predicted to form a fusion protein, P1-P2 (152 kDa), involved in virus replication.

The sequence-based virus classification tool (1) showed 95.3% nucleotide identity of the full-genome sequence of AEV-2 to the genome sequence of a recently discovered alfalfa enamovirus 1 (AEV-1, GenBank accession no. NC_029993), a new member of the genus *Enamovirus*, family *Luteoviridae*, associated with alfalfa dwarfism disease in Argentina (2), and 75.2% identity to pea enation mosaic virus 1 (PeMV-1), the type species of the genus *Enamovirus* (GenBank accession no. NC_003629).

On the amino acid level (BLASTp), AEV-2 ORF0 is 98% and 70% identical to the ORFs encoding hypothetical proteins (HPs) of AEV-1 (query coverage, 100%; E value, 0.0; GenBank accession no. YP_009249822.1) and PeMV-1 (query coverage, 96%; E value, 1e-142; GenBank accession no. AD086938.1), respectively. Predicted protein encoded by AEV-2 ORF1 is 97% identical to the HP of AEV-1 (query coverage, 100%; E value, 0.0; GenBank accession no. YP_009249824.1) and 71% identical to the HP of PeMV-1 (query coverage, 100%; E value, 0.0; GenBank accession no. AD086939.1). Putative ORF2 product is 95% identical to the RdRp protein of AEV-1 (query coverage, 99%; E value, 0.0; GenBank accession no. YP_009249823.1) and 88% identical to the polymerase protein of PeMV-1 (query coverage, 100%; E value, 0.0; GenBank accession no. AAA72297.1). AEV-2 CP (ORF3) is 96% and 78% identical to the CP of AEV-1 (query coverage, 100%; E value, 5e-114; GenBank accession no. YP_009249826.1) and CP of PeMV-1 (query coverage, 100%; E value, 4e-98; GenBank accession no. CAA70314.1), respectively. Projected ATS of AEV-2 (ORF5) is 94% identical to the tentative ATS of AEV-1 (query coverage, 100%; E value, 0.0; GenBank accession no. YP_009249825.1) and 71% identical to the ATS of PeMV-1 (query coverage, 99%; E value, 0.0; GenBank accession no. CAA70315.1).

Phylogenetic analyses based on the alignments of the predicted RdRp amino acid sequences and the complete nucleotide sequences of AEV-2 with other members of the family* Luteoviridae* grouped AEV-2 in the same cluster with AEV-1. Thus, we propose that AEV-2 represents a Sudanese strain of AEV-1. Although the exact origin of alfalfa enamovirus is unknown, it is likely that its evolution and dissemination into new areas are linked to the host geography. The first incidence of the virus in a different location (Sudan) suggests that it might be widespread and can also occur in Iran and southwestern Asia, the geographic origin of alfalfa (3), as well as in other alfalfa cultivation regions worldwide.

### Accession number(s).

The complete genomic sequence of AEV-2 has been deposited in GenBank under accession number KY985463.
